# Parenteral Vaccination with a Live *Brucella melitensis* Mutant Protects against Wild-Type *B. melitensis* 16M Challenge

**DOI:** 10.3390/microorganisms12010169

**Published:** 2024-01-15

**Authors:** Xinghong Yang, Zakia I. Goodwin, Ella Bhagyaraj, Carol Hoffman, David W. Pascual

**Affiliations:** Department of Infectious Diseases & Immunology, College of Veterinary Medicine, University of Florida, Gainesville, FL 32611, USA; yangxh@ufl.edu (X.Y.); zakiagoodwin721@gmail.com (Z.I.G.); bhagyaraj970@gmail.com (E.B.); riccardic@ufl.edu (C.H.)

**Keywords:** *Brucella*, T cells, IFN-γ, TNF-α, vaccine

## Abstract

Susceptibility to brucellosis remains prevalent, even in herds vaccinated with conventional vaccines. Efforts are underway to develop an improved brucellosis vaccine, and possibly a universal vaccine, given that *Brucella* species are highly homologous. To this end, two *B. melitensis* mutants were developed, znBM-*lacZ* (znBMZ) and znBM-mCherry (znBM-mC), and were tested for their ability to confer systemic immunity against virulent *B. melitensis* challenge. To assess the extent of their attenuation, bone-marrow-derived macrophages and human TF-1 myeloid cells were infected with both mutants, and the inability to replicate within these cells was noted. Mice infected with varying doses of znBM-mC cleared the brucellae within 6–10 weeks. To test for efficacy against systemic disease, groups of mice were vaccinated once by the intraperitoneal route with either znBMZ or *B. abortus* S19 vaccine. Relative to the PBS-dosed mice, znBMZ vaccination greatly reduced splenic brucellae colonization by ~25,000-fold compared to 700-fold for S19-vaccinated mice. Not surprisingly, both znBMZ and S19 strains induced IFN-γ^+^ CD4^+^ T cells, yet only znBMZ induced IFN-γ^+^ CD8^+^ T cells. While both strains induced CD4^+^ effector memory T cells (Tems), only znBMZ induced CD8^+^ Tems. Thus, these results show that the described znBM mutants are safe, able to elicit CD4^+^ and CD8^+^ T cell immunity without a boost, and highly effective, rendering them promising vaccine candidates for livestock.

## 1. Introduction

*Brucella* species are Gram-negative, intracellular bacteria [1,2] that are mostly considered a disease of livestock resulting in the abortion of offspring [3,4,5,6,7]. Brucellosis poses a socio-economic burden for many countries [7,8,9,10,11,12]. Specific *Brucella* species show a propensity for infecting livestock. Cattle is mostly infected by *Brucella abortus*; goats and sheep are infected by *B. melitensis*; pigs are infected by *B. suis*; and sheep can be infected by *B. ovis* [10,11]. Zoonosis, primarily from infection with *B. abortus*, *B. melitensis*, or *B. suis*, produces a debilitating chronic disease that causes undulant fever and fatigue in patients and can lead to the involvement of various organ systems if left untreated [7,8,13,14]. In fact, brucellosis is the third most neglected disease worldwide [15]. Humans acquire disease primarily via the consumption of tainted, e.g., unpasteurized, dairy products or via aerosol exposure [7,13,14]. If diagnosis is made early, antibiotic treatment can eradicate infection; however, treatment requires daily dosing with two or three antibiotics for six weeks and, in some cases, treatment does not eradicate the infection [8,13,14]. Treated patients may still have up to a 16% chance for remaining infected [13]. No vaccine for human brucellosis is currently available [7,13,14].

Vaccines for livestock are available and widely used, but these are not completely effective in preventing or treating disease [3,4,7]. Protection from brucellosis requires the stimulation of cell-mediated immunity, primarily via T helper 1 (Th1) cells producing IFN-γ and TNF-α [16,17,18,19,20,21]. Past work in experimental systems, as well as in relevant livestock, show that protection requires Th1-type immunity [2,4,7,22,23,24]. In fact, humans with an IFN-γ deficiency show an increased likelihood of chronic disease due to the inability to prevent *Brucella*’s dissemination [25,26,27]. Natural infections and livestock vaccines do stimulate antibody (Ab) responses, albeit primarily directed against *Brucella*’s LPS, yet these Abs are unable to confer protection [19,28]. Thus, an effective vaccine must be able to elicit strong Th1 cell immunity.

Next-generation brucellosis vaccines are needed to improve livestock health and reduce zoonotic transmission. Effectively vaccinating livestock would reduce disease transmission to humans [29]. To this end, our laboratory has generated various *Brucella* mutants based on the deletion of their *znuA*, which is responsible for zinc uptake [30,31,32]. The deletion of *znuA* greatly reduces *Brucella*’s virulence [30,31]. To address THE possible reversion to a wild-type (wt) phenotype, a second mutation was introduced, interrupting their *norD* which encodes for nitric oxide reductase [33]. These combined mutations resulted in the development of attenuated ∆*znuA* ∆*norD B. abortus-lacZ* (znBAZ; [34,35]) and ∆*znuA* ∆*norD B. melitensis*-mCherry (znBM-mC; [36]) strains. As described here, the additional mutant, ∆*znuA* ∆*norD B. melitensis*-*lacZ* (znBMZ), was also generated. Previous work with znBAZ showed that two doses, administered parenterally [34] or mucosally [35], were required for optimal protection against wt challenge with virulent *B. abortus* 2308. Mice mucosally dosed twice with znBM-mC were optimally protected against wt challenge with virulent *B. melitensis* 16M [36]. Herein, the objective of the current study was to ascertain whether the route of znBMZ vaccination influences the type of immunity elicited, and whether a single intraperitoneal (IP) dose can confer potent protection against parenteral challenge with wt *B. melitensis* 16M.

## 2. Materials and Methods

### 2.1. Brucella Strains

The development of the live attenuated *Brucella melitensis* mutant, znBM-mC, which lacks *znuA* and *norD* and expresses mCherry, was previously described [36]. The znBMZ strain was constructed similar to znBM-mC, but the *E. coli lacZ* was incorporated into the uncoded regions between BMEI1800 and BMEI1801. The smooth *B. melitensis* Rev 1 livestock vaccine was used as previously described [37], and wt *B. melitensis* 16M was obtained through BEI Resources, NIAID, and NIH. The smooth *B. abortus* S19 was acquired as previously described [31]. All *Brucella* strains were grown on potato infusion agar (PIA) plates for 3 days at 37 °C under 5% CO_2_. Brucellae were harvested with sterile Dulbecco’s phosphate-buffered saline (DPBS; Life Technologies Corp., Grand Island, NY, USA), washed twice, and diluted with DPBS for use.

### 2.2. Mice

Female BALB/c mice at 6–8 weeks age were acquired from Charles River Laboratory (Frederick, MD, USA). All animal experiments using the live attenuated *Brucella* strains S19, znBMZ, or znBM-mC were conducted under biosafety level-2 (BSL-2) containment. Those studies involving wt *B. melitensis* 16M or *B. melitensis* Rev 1 vaccine were performed under BSL-3 containment. Mice were maintained in individually ventilated cages under HEPA-filtered barrier conditions with 12 h of light and 12 h of darkness, and food and water were provided ad libitum. All animal care and procedures were in strict accordance with the recommendations in the Guide for the Care and Use of Laboratory Animals of the National Institutes of Health. All animal studies were conducted under protocols approved by the University of Florida Institutional Animal Care and Use Committee.

### 2.3. Evaluation of znBMZ’s and znBM-mC’s Attenuation in Macrophages

Naive mice were humanely euthanized to procure their femurs and tibias. The long bones were flushed with 5 mls of complete media (CM) using a sterile syringe and a 25 gauge needle. CM consisted of RPMI-1640 (Caisson Labs, Inc., Smithfield, UT, USA) plus various supplements (Caisson Labs) including 10 mM HEPES buffer, 10 mM penicillin/streptomycin, 10 mM nonessential amino acids, and 10 mM sodium pyruvate plus 10% fetal bovine serum (FBS; Atlanta Biologicals, Norcross, GA, USA). Bone marrow cells (2 × 10^6^/mL) were subjected to differentiation using mouse 25 ng/mL M-CSF (Peprotech, Cranberry, NJ, USA) for 7 days with new media containing M-CSF added on day 3. On day 7, adherent cells were gently scraped and washed in antibiotic-free incomplete media (ICM). Antibiotic-free ICM consisted of RPMI-1640 and 10 mM HEPES buffer. Flow cytometry analysis revealed that >96% of these adherent cells were bone-marrow-derived macrophages (BMDMs) by their dual expression of F4/80 and CD11b.

BMDMs and human TF-1 myeloid cells (American Type Culture Collection, Manassas, VA) were seeded into 96-well microtiter plates (Falcon Corning, Glendale, AZ, USA) at 1 × 10^4^ cells/mL overnight in antibiotic-free ICM to allow to adhere. BMDMs and TF-1 cells were then infected with wt *B. melitensis* 16M, *B. melitensis* Rev 1, znBMZ, or znBM-mC at 10:1 (bacteria:cell) for 4 h, then cells were washed twice with CM and incubated with CM containing 50 μg/mL gentamicin (Life Technologies-Invitrogen, Carlsbad, CA, USA) for 30 min to rid cultures of extracellular bacteria. Cells were then cultured in CM without antibiotics for the indicated time points: 0, 24, 48, and 72 h. At each time point, samples were lysed in 100 μL of sterile water. Lysate dilutions were then plated on PIA plates. The PIA plates were incubated at 37 °C under 5% CO_2_ for three days. Brucellae colonies were then enumerated.

### 2.4. Assessment of znBM-mC’s Attenuation In Vivo

*B. abortus* S19 and znBM-mC were grown overnight on PIA for 2–3 days at 37 °C, 5% CO_2_. Bacteria were washed twice in sterile DPBS, resuspended in DPBS, and dose estimated by spectrophotometric measurement at OD600. Bacteria were diluted in DPBS for an appropriate dose in 200 µL suspension, which was then given to mice by the intraperitoneal (IP) route. The actual viable inoculum was confirmed by serial dilution tests on PIA. To measure the extent of in vivo tissue colonization, various dilutions of znBM-mC were administered: 10^5^, 10^6^, or 10^7^ CFUs znBM-mC or 10^6^ CFUs *B. abortus* S19 vaccine in 200 µL DPBS were given IP to BALB/c mice. Splenic weights and CFU counts were assessed at 2, 4, 6, and 10 weeks post-infection. Individual spleens were mechanically homogenized in 1.0 mL of sterile Milli-Q water. Various dilutions of splenic homogenates were incubated for 3–5 days at 37 °C in 5% CO_2_ on PIA, after which bacterial colonies were enumerated and calculated CFUs per spleen were determined [31].

### 2.5. Evaluation of znBMZ’s Efficacy following Parenteral Vaccination

Groups of BALB/c female mice were IP vaccinated with DPBS vehicle (n = 4), 1 × 10^7^ CFUs S19 (n = 7), or 1 × 10^7^ CFUs znBMZ (n = 6) on day 0. On day 42, all mice were IP challenged with 5 × 10^4^ CFUs wt *B. melitensis* 16M in 200 μL DPBS. Spleens from individual mice were harvested four weeks later, weighed, and evaluated for brucellae colonization on PIA plates with X-gal.

### 2.6. Evaluation of Cellular Immunity and Cytokine Analysis

Groups of mice (5/group) were IP vaccinated with znBMZ, S19, or DPBS, as described above, and evaluated 42 days after vaccination. Ex vivo intracellular IFN-γ expression by immune T cells were measured by flow cytometry similarly to previously described [35,36]. Lymphocytes were then stained for TCRβ, CD4, CD8 T cell, CD44, CX3CR1, and KLRG1 mAbs (BioLegend, San Diego, CA, USA; eBioscience, San Diego, CA, USA), washed, and then fixed with IC Fixation Buffer (eBioscience). Subsequently, cells were permeabilized with Permeabilization Buffer (eBioscience), and stained with labeled anti-IFN-γ (eBioscience). Fluorescence was acquired on Cytek Auora flow cytometer using SpectroFlo^®^ software version 2.0 (Fremont, CA, USA). All samples were analyzed using FlowJo software version 10.8.1 (Tree Star, Ashland, OR, USA).

To measure soluble proinflammatory cytokine production by each treatment group, isolated splenic mononuclear cells (3 × 10^6^) in 1.0 mL of CM were restimulated with 10^9^ CFUs of heat-killed RB51 (HKRB51) at 37 °C under 5% CO_2_. After three days of in vitro stimulation, culture supernatants were harvested for cytokine analyses. IFN-γ, TNF-α, and IL-17 were measured by cytokine-specific sandwich-based ELISAs using the relevant monoclonal Abs for detection as previously described [37,38].

### 2.7. Statistical Analysis

The statistical differences were calculated using the Tukey–Kramer Multiple Comparisons Test to measure differences among variations of in vitro infection and in vivo splenic weight, as well as colonization by znBMZ, znBM-mC, *B. abortus* S19, or wt *B. melitensis* 16M at the 95% confidence interval. One-way analysis of variance (ANOVA) was used to compare differences in the types of T cell and cytokine responses. SigmaPlot version 10.0 and SigmaStat version 3.5 (Systat Software, Palo Alto, CA, USA) were used for determining significance and generating graphs.

## 3. Results

### 3.1. znBMZ and znBM-mC Are Attenuated in Mouse Macrophages and Human Myeloid Cells

A past study examined the suitability of znBM-mC as a vaccine prototype when administered mucosally [36]. In addition, the znBMZ strain was generated to be similar to its related znBM-mC strain [36]. The current work examines the effectiveness in stimulating host immunity to *B. melitensis* subsequent to parenteral vaccination.

**Figure 1:**
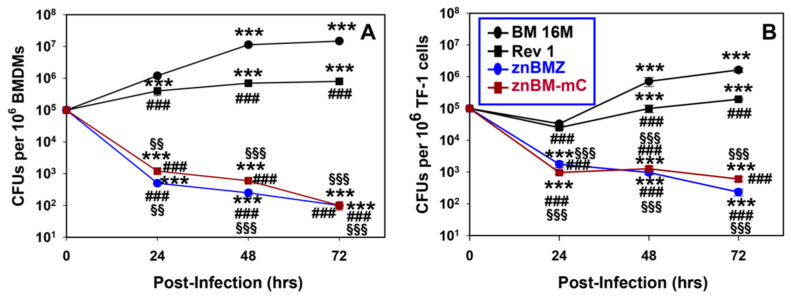
Two recombinant strains, ∆*znuA* ∆*norD B. melitensis-lacZ* (znBMZ) and ∆*znuA* ∆*norD B. melitensis*-mCherry (znBM-mC), show reduced growth in (**A**) primary mouse bone-marrow-derived macrophages (BMDMs) and (**B**) human TF-1 myeloid cells. (**A**) Wild-type (wt) *B. melitensis* 16M (BM 16M), *B. melitensis* Rev 1 vaccine, znBMZ, and znBM-mC were used to infect BMDMs (1 × 10^4^/well) at a bacteria-to-macrophage ratio of 10:1. Primary BMDMs were derived from BALB/c mouse femurs and recovered BMDMs were found to be >96% CD11b^+^ F4/80^+^. After a 3-h incubation followed by a 30 min treatment with 50 μg/mL gentamicin to rid extracellular brucellae, infected BMDMs were incubated in fresh medium for 0, 24, 48, or 72 h. Infected macrophages were water-lysed, and supernatants were diluted for CFU enumeration on potato infusion agar (PIA). The level of initial infection was the same for all *Brucella* strains (t = 0 h). The results show that both znBMZ and znBM-mC were unable to achieve the level of colonization as the wt BM 16M strain or *B. melitensis* Rev 1 vaccine. (**B**) Human TF-1 myeloid cells (1 × 10^4^/well) were infected 10:1 with wt BM 16M, *B. melitensis* Rev 1 vaccine, znBMZ, or znBM-mC, and evaluated for colonization as described above. The results showed that both znBMZ and znBM-mC were unable to achieve the same level of colonization as wt BM 16M or Rev 1. Values are the means of triplicate wells ± SEM: *** *p* ≤ 0.002; differences in brucellae colonization by the same strain over time vs. t = 0; ^###^ *p* ≤ 0.001; differences in brucellae colonization vs. wt BM 16M at the same time point; and ^§§^ *p* < 0.01, ^§§§^ *p* < 0.001 differences in brucellae colonization vs. BM Rev 1 vaccine.

To determine if znBMZ and znBM-mC are attenuated in primary macrophages, BMDMs were generated, and infected at a 10:1 (brucellae to macrophage) ratio. Macrophages were water-lysed at 24, 48, and 72 h after infection to determine the extent of colonization by znBMZ and znBM-mC relative to wt *B. melitensis* 16M and *B. melitensis* Rev 1 strains (Figure 1A). Macrophage colonization by both znBMZ and znBM-mC progressively diminished over time up to 72 h post-infection, and relative to wt *B. melitensis* 16M and Rev 1 vaccine. In fact, both wt *B. melitensis* and the Rev 1 vaccine showed a progressively increased colonization of the BMDMs over time, although Rev 1 colonized to a lesser extent.

The colonization capacity in human myeloid TF-1 cells was assessed using similar infection ratios with wt and *Brucella* mutants as carried out for the BMDMs. Again, both wt *B. melitensis* 16M and *B. melitensis* Rev 1 strains showed a significantly increased colonization of TF-1 cells at 48 and 72 h post-infection (Figure 1B). In contrast, both znBMZ and znBM-mC showed diminished CFUs throughout the three time points with a progressive decline in colonization. Thus, this evidence demonstrates that both znBMZ and znBM-mC are attenuated, and can be cleared from mouse and human cells.

### 3.2. znBM-mC Is Attenuated In Vivo

**Figure 2:**
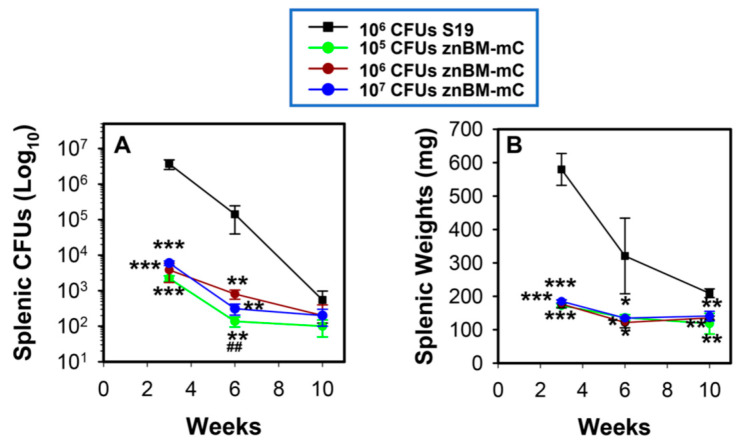
The *Brucella* mutant, znBM-mC, is attenuated since it is effectively cleared from mice following intraperitoneal (IP) delivery. BALB/c mice (4–7/group/time point) were IP vaccinated with 1 × 10^6^ CFUs of *B. abortus* S19 or with the indicated dose of znBM-mC (10^5^, 10^6^, or 10^7^ CFUs). At 3, 6, and 10 weeks (wks) after vaccination, individual spleens were assessed for (**A**) brucellae colonization and (**B**) splenic weights. Values are the mean CFUs or weights from individual mice ± SEM; *** *p* < 0.001, ** *p* ≤ 0.02, * *p* < 0.05 differences vs. S19 colonization at the specific time point; ^##^ *p* = 0.018 between mice dosed with 10^5^ or 10^6^ CFUs of znBM-mC at 6 wks post-infection.

To assess the persistence of znBM-mC’s in vivo, a colonization study was performed. Groups of BALB/c mice were tested with three IP doses of znBM-mC: 1 × 10^5^, 1 × 10^6^, and 1 × 10^7^ CFUs. These were compared to mice dosed with 1 × 10^6^ CFUs S19. Individual mice were measured for splenic colonization (Figure 2A) and changes in splenic weights (Figure 2B) at 3, 6, and 10 weeks post-infection. All three of the groups dosed with

znBM-mC showed a progressive decline in splenic colonization, which was significantly less than that in mice infected with S19. The clearance of znBM-mC did not appear to be dose-dependent, since only one time point showed a significant difference between groups infected with 1 × 10^5^ or 1 × 10^6^ CFUs at 6 weeks post-infection (Figure 2A). Spleen weights from the znBM-mC-infected mice were significantly less than the S19-infected group, and the dose of znBM-mC did not affect splenic weights at any time point measured (Figure 2B). These data show that znBM-mC is attenuated in vivo following IP infection.

### 3.3. IP Vaccination of BALB/c Mice with znBMZ Confers Immune Protection

**Figure 3:**
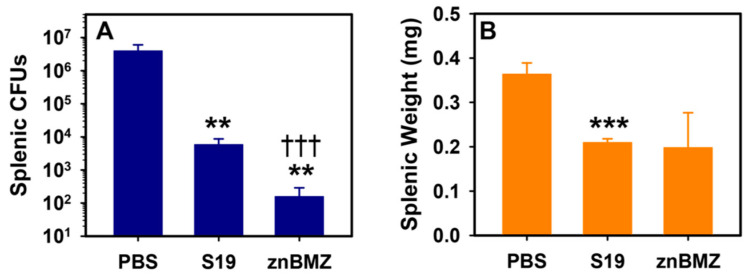
A single IP vaccination with znBMZ protects mice against wt *Brucella* colonization. Groups of BALB/c mice were IP vaccinated with sterile PBS vehicle (n = 4), 10^7^ CFUs S19 (n = 7), or 10^7^ CFUs znBMZ (n = 6) on day 0. On day 42, all mice were challenged IP with 5 × 10^4^ CFUs wt BM 16M. Spleens from individual mice were harvested and evaluated (**A**) for brucellae colonization on PIA plates with X-gal and (**B**) for individual weights. Values are the mean CFUs or weights from individual mice ± SEM; *** *p* < 0.001, ** *p* ≤ 0.01, differences vs. PBS-treated mice; ^†††^ *p* < 0.001 vs. S19-vaccinated mice.

To measure znBMZ’s ability to induce protective immunity, groups of BALB/c mice were IP vaccinated with 1 × 10^7^ CFUs S19 or znBMZ, and compared to mice dosed with DPBS vehicle. Six weeks after vaccination, all mice were subjected to an IP challenge with 5 × 10^4^ CFUs of wt *B. melitensis* 16M, and four weeks post-challenge, individual spleens were harvested for colonization and weights. The *B. abortus* S19 reduced splenic colonization by ~676-fold, but znBMZ proved more effective by reducing colonization by more than 25,000-fold (Figure 3A). Such a reduction by the znBMZ-vaccinated mice was significantly different from the PBS-treated and S19-immunized mice. None of the colonies detected from the znBMZ-vaccinated spleens showed a blue color, indicating that the white colonies present were wt *B. melitensis* 16M. Although both S19- and znBMZ-vaccinated mice showed less splenic inflammation than the PBS-treated mice, the splenic weights between the two vaccinated groups were not significantly different (Figure 3B).

### 3.4. IP Vaccination with znBMZ Stimulates Induction of CD4^+^ and CD8^+^ T Effector Memory Cells (Tems)

**Figure 4:**
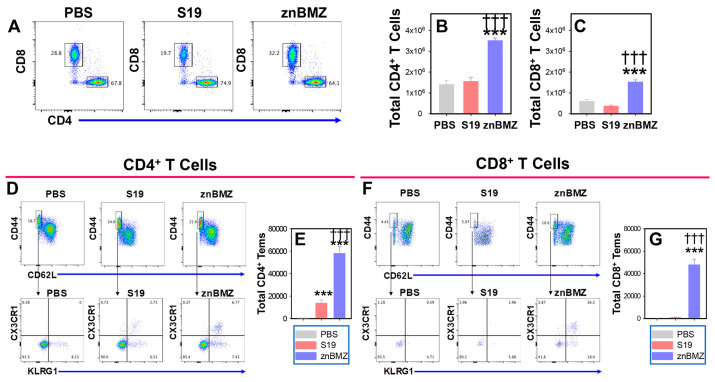
IP vaccination with znBMZ induces CD4^+^ and CD8^+^ T effector memory cells (Tems). Groups of BALB/c mice were IP vaccinated with sterile PBS vehicle (n = 5), 10^7^ CFUs S19 (n = 5), or 10^7^ CFUs znBMZ (n = 5) on day 0. On day 42, spleens from individual mice were harvested and mononuclear cells were isolated for evaluation T cell subsets by flow cytometry. (**A**) Flow cytometry plots reveal that S19-vaccinated mice elicit mostly CD4^+^ T cells in contrast to both CD4^+^ and CD8^+^ T cells induced with subsequent znBMZ vaccination. (**B**) Total CD4^+^ and (**C**) CD8^+^ T cells are depicted. The frequencies of (**D**) CD4^+^ and (**F**) CD8^+^ T cells were evaluated for induction of Tems by staining splenic lymphocytes for CD44^High^ CD62^Low^ CX3CR1^+^ KLRG1^+^ T cells and (**E**) total CD4^+^ and (**G**) CD8^+^ Tems were quantified. Values are the mean total T cells and Tems from individual mice ± SEM; *** *p* < 0.001, differences vs. PBS-treated mice; ^†††^ *p* < 0.001 vs. S19-vaccinated mice.

To determine the types of T cell responses induced following IP vaccination, groups of BALB/c mice were IP vaccinated with 10^7^ CFUs S19 or znBMZ on day 0, and spleens were harvested 42 days later. Stained splenic mononuclear cells were gated on TCRβ^+^ lymphocytes (Figure 4A) and were assessed for total CD4^+^ and CD8^+^ T cells (Figure 4B,C). Vaccination with znBMZ resulted in greater numbers of CD4^+^ and CD8^+^ T cells than those obtained with S19-vaccinated mice or age-matched naive (PBS-dosed) mice. A further evaluation of the induced CD44^High^ memory T cells revealed that the frequency of Tem induction, defined as CD44^High^ CD62^Low^ CX3CR1^+^ KLRG1^+^, was increased in znBMZ-vaccinated mice (Figure 4D,F). The total CD4^+^ and CD8^+^ Tems were significantly increased in the znBMZ-vaccinated mice (Figure 4E,G), particularly for the CD8^+^ Tems (Figure 4G). The S19-vaccinated mice showed no significant change from the PBS-dosed mice with regard to their CD8^+^ Tems.

### 3.5. IP Vaccination with znBMZ Stimulates IFN-γ Production

**Figure 5:**
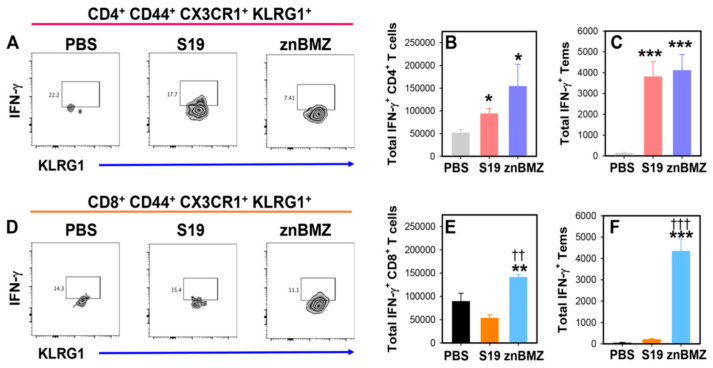
IP vaccination with znBMZ induces IFN-γ-producing CD4^+^ and CD8^+^ T cells. The spleens from the same groups of mice evaluated in Figure 4 were further analyzed for IFN-γ^+^ T cells by flow cytometry. (**A**) Gating on CD4^+^ Tems, these were further analyzed for expression of IFN-γ. (**B**) The total number of IFN-γ^+^ CD4^+^ T cells and (**C**) IFN-γ^+^ Tems are depicted. (**D**) Gating on CD8^+^ Tems, these were further analyzed for expression of IFN-γ. (**E**) The total number of IFN-γ^+^ CD8^+^ T cells and (**F**) IFN-γ^+^ Tems are shown. Values are the mean total CD4^+^ and CD8^+^ T cells and CD4^+^ and CD8^+^ Tems from individual mice ± SEM; *** *p* < 0.001, ** *p* ≤ 0.002, * *p* < 0.05 differences vs. PBS-treated mice; ^†††^ *p* < 0.001, ^††^ *p* ≤ 0.002 vs. S19-vaccinated mice.

The same splenic lymphocytes from Figure 4 were further evaluated for the source of IFN-γ production. Splenic lymphocytes from individual mice were stained for surface T cell molecules and subjected to intracellular staining for IFN-γ. Although the frequency of IFN-γ^+^ CD4^+^ or CD8^+^ Tems did not significantly differ among the groups (Figure 5A,D), the total IFN-γ^+^ CD4^+^ T cells were significantly increased for both S19- and znBMZ-vaccinated mice (Figure 5B). Only the total IFN-γ^+^ CD8^+^ T cells were significantly increased for znBMZ-vaccinated mice (Figure 5E). A further evaluation showed that both S19- and znBMZ-vaccinated mice had significantly increased total IFN-γ^+^ CD4^+^ Tems (Figure 5C). Only the znBMZ-vaccinated mice exhibited increased IFN-γ^+^ CD8^+^ Tems (Figure 5F).

**Figure 6:**
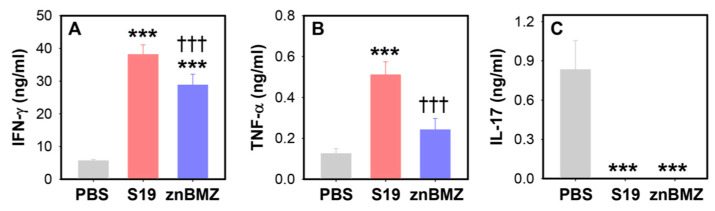
A single IP vaccination with S19 or znBMZ elicits increased splenic IFN-γ and TNF-α, but no IL-17 following in vitro restimulation. Isolated splenic mononuclear cells from individual mice, described in Figure 4 and Figure 5, were restimulated in vitro with media or 10^9^ CFUs HKRB51 for three days. Harvested supernatants from duplicate cultures were analyzed for (**A**) IFN-γ, (**B**) TNF-α, and (**C**) IL-17. Values are the mean cytokines from duplicate cultures corrected for media only levels from individual mice ± SEM; *** *p* < 0.001, differences vs. PBS-treated mice; ^†††^ *p* < 0.001 vs. S19-vaccinated mice.

To assess whether other cytokines are induced by IP vaccination with the attenuated *Brucella* strains, the splenic lymphocytes from the same groups of mice were restimulated with HKRB51 for three days, and harvested supernatants were measured for IFN-γ, TNF-α, and IL-17 production (Figure 6). Both S19 and znBMZ produced increased levels of IFN-γ and TNF-α (Figure 6A,B). Neither showed stimulation for IL-17 (Figure 6C).

## 4. Discussion

Available livestock vaccines have proved effective in maintaining brucellosis-free herds when combined with test and slaughter interventions [7,12,39]. However, not all countries can afford to compensate producers for slaughtered animals, and many producers are reliant on animals for dairy and meat [10,11,40]. Thus, a highly effective brucellosis vaccine is needed. To remedy this shortcoming, efforts are underway to develop improved livestock vaccines, and possibly vaccination regimens [7]. With respect to the latter, mucosal vaccinations, which can improve immunity at sites where invasion occurs, are worth consideration [7].

To ascertain its effectiveness for vaccination, the IP route was selected since it better mimics disease outcome resulting in a systemic disease [6,7]. Moreover, IP vaccination has proven effective in experimental models in conferring protection and significant brucellae clearance [41]. A limitation with conventional *Brucella* vaccines lies in their incomplete efficacy, providing only about 70% efficacy against wt *Brucella* abortion challenges [11,42]. Additionally, due to their residual virulence, these vaccines pose risks to humans via inadvertent exposure [7,43,44]. Even the rough vaccine, RB51, remains infectious to heifers and their fetuses [45], and to humans via contaminated milk [46].

To improve upon these limitations, our efforts are focused on developing an efficacious vaccine with minimal residual virulence. For nearly two decades, our lab has focused on developing mutants based on zinc uptake mechanisms for both *B. abortus* and *B. melitensis* [31,34,35,36,37,38,47]. *Brucella* deficient in *znuA* showed considerable attenuation, but its elimination from the host took considerable time, proving more effective when administered by the mucosal [37,47] rather than the parenteral route [31]. However, the introduction of a second mutation, ∆*norD*, resulted in improved attenuation for znBAZ [34], as shown here for znBMZ and znBM-mC. The level of attenuation by znBMZ and znBM-mC in primary mouse macrophages and human TF-1 myeloid cells is similar to that observed for znBAZ in mouse RAW264.7 macrophages and human peripheral blood macrophages [34]. znBM-mC is also attenuated in vivo, as evidenced by its clearance from the host spleen between 6 and 10 weeks after IP vaccination. Its persistence is similar to znBAZ, which cleared by 8 weeks post-IP vaccination [34].

Upon establishing the attenuation of the ∆*znuA* ∆*norD B. melitensis* mutants, subsequent analysis examined the efficacy of this double mutation. When using a single IP dose of 1 × 10^7^ CFUs for both, znBMZ conferred better protection than the S19 vaccine. Interestingly, when compared to IP znBAZ-vaccinated mice [34], a single dose of znBMZ proved equivalent to two doses of znBAZ. Perhaps the znBM mutants are slightly more virulent or subtle differences between *B. abortus* and *B. melitensis* in pathogenicity may account for the improved efficacy by znBMZ. More testing in relevant livestock is needed to ascertain znBM mutants’ capability to protect in different situations.

An evaluation of the type of T cell immunity elicited by znBMZ vaccination revealed that IP vaccination induced both CD4^+^ and CD8^+^ T cell responses with total T cell numbers being greater than those obtained with S19-vaccinated mice. S19 vaccination exhibited a predilection for stimulating CD4^+^ T cells, rather than CD8^+^ T cells, similar to that observed following a single nasal dose of our *B. abortus* mutant, znBAZ [38]. It was surprising to observe the stimulation of CD8^+^ T cells, since only a single parenteral vaccination was administered in contrast to past mucosal vaccinations [35,36,37,38,47]. Such a finding suggests that the stimulation of CD8^+^ T cells may be dependent on the *Brucella*’s mutations, and less upon the route of administration. Further inquiry into the type of memory T cells elicited by znBMZ vaccination revealed CD44^High^ CD62^Low^ CX3CR1^+^ KLRG1^+^ Tems being induced [48,49]. The presence of resident memory T cells was not evident in the spleen, nor was it anticipated. Given that mucosal vaccinations were not performed [35,36], it seemed unlikely that we would detect these. Since IFN-γ is essential for protection against *Brucella* infections [7,11,17,18,19], the source of IFN-γ was investigated. Vaccination with either S19 or znBMZ elicited equivalent levels of IFN-γ-producing total CD4^+^ T cells and total CD4^+^ Tems. How they differed was associated with the stimulation of CD8^+^ T cells. Vaccination with znBMZ resulted in the stimulation of elevated IFN-γ-producing total CD8^+^ T cells and total CD8^+^ Tems. In fact, vaccination with S19 resulted in no significant difference in the number of IFN-γ-producing total CD8^+^ T cells or total CD8^+^ Tems from PBS-dosed mice, which is consistent with past observations [38]. Thus, vaccination with znBMZ had the additional benefit of inducing IFN-γ^+^ CD8^+^ Tems. An evaluation of the whole splenic mononuclear cell production of IFN-γ following in vitro antigen restimulation showed S19-vaccinated mice producing more IFN-γ than spleens from znBMZ-vaccinated mice. Although the difference between the two groups was subtle, it was significant, and both groups did respond favoring IFN-γ production. However, protection was more efficient with the znBMZ-vaccinated mice, suggesting that other mechanisms may be contributing to such protection, possibly via CD8^+^ T cell effector mechanisms. Although we were unable to detect TNF-α by intracellular cytokine staining, spleens from S19-vaccinated mice showed greater TNF-α-producing capacity than spleens from znBMZ-vaccinated mice. IL-17 was undetectable for either S19- or znBMZ-vaccinated groups. Thus, the difference in protection between the two groups is neither TNF-α- nor IL-17-dependent. Regarding the latter, IL-17 induction may be more dependent on mucosal routes of vaccination, as it was found to be induced by the various ∆*znuA Brucella* mutants [35,36,37,38,47].

In summary, the development of znBMZ and znBM-mC shows these mutants to be highly protective against systemic challenge with virulent wt *B. melitensis* 16M. Even a single dose proved to be strikingly effective. These mutants lack residual virulence, which will be important for testing in relevant livestock.

## Data Availability

Data are contained within the article.
